# Are *k13* and *plasmepsin II* genes, involved in *Plasmodium falciparum* resistance to artemisinin derivatives and piperaquine in Southeast Asia, reliable to monitor resistance surveillance in Africa?

**DOI:** 10.1186/s12936-019-2916-6

**Published:** 2019-08-23

**Authors:** Francis Foguim Tsombeng, Mathieu Gendrot, Marie Gladys Robert, Marylin Madamet, Bruno Pradines

**Affiliations:** 1 Unité Parasitologie et Entomologie, Département Microbiologie et maladies infectieuses, Institut de Recherche Biomédicale des Armées, 19-21 Boulevard Jean Moulin, 13005 Marseille, France; 2 Aix Marseille Univ, IRD, SSA, AP-HM, VITROME, Marseille, France; 3 IHU Méditerranée Infection, Marseille, France; 4 Centre National de Référence du Paludisme, Institut de Recherche Biomédicale des Armées, Marseille, France

**Keywords:** Malaria, *Plasmodium falciparum*, Anti-malarial drug, Resistance, In vitro, Dihydroartemisinin, Piperaquine, Plasmepsin II, K13

## Abstract

Mutations in the propeller domain of *Plasmodium falciparum kelch 13* (*Pfk13*) gene are associated with artemisinin resistance in Southeast Asia. Artemisinin resistance is defined by increased ring survival rate and delayed parasite clearance half-life in patients. Additionally, an amplification of the *Plasmodium falciparum plasmepsin II* gene (*pfpm2*), encoding a protease involved in hemoglobin degradation, has been found to be associated with reduced in vitro susceptibility to piperaquine in Cambodian *P. falciparum* parasites and with dihydroartemisinin–piperaquine failures in Cambodia. The World Health Organization (WHO) has recommended the use of these two genes to track the emergence and the spread of the resistance to dihydroartemisinin–piperaquine in malaria endemic areas. Although the resistance to dihydroartemisinin–piperaquine has not yet emerged in Africa, few reports on clinical failures suggest that *k13* and *pfpm2* would not be the only genes involved in artemisinin and piperaquine resistance. It is imperative to identify molecular markers or drug resistance genes that associate with artemisinin and piperaquine in Africa. *K13* polymorphisms and *Pfpm2* copy number variation analysis may not be sufficient for monitoring the emergence of dihydroartemisinin–piperaquine resistance in Africa. But, these markers should not be ruled out for tracking the emergence of resistance.

## Background

According to the World Health Organization (WHO), artemisinin-based combination therapy (ACT) has been recommended as treatment of uncomplicated falciparum malaria since 2001. However, *Plasmodium falciparum* parasites resistant to artemisinin derivatives emerged in Southeast Asia, and more particularly in western Cambodia, Myanmar, Thailand and Laos [1–6]. More recently, the emergence of *P. falciparum* resistance to dihydroartemisinin–piperaquine was observed in Cambodia, where recrudescent infections had rapidly increased [7–9], and then in Vietnam [10, 11]. However, dihydroartemisinin–piperaquine is little-used in African countries for the treatment of uncomplicated malaria, where artemether–lumefantrine and/or artesunate–amodiaquine are currently used. Only Senegal has adopted dihydroartemisinin–piperaquine as a third alternative first-line regimen. Dihydroartemisinin–piperaquine has emerged as a potential combination for chemoprevention in pregnant women and children in Africa [12–14].

According to the WHO, the resistance to dihydroartemisinin–piperaquine has not yet emerged in Africa. There is currently no evidence of failing efficacy of dihydroartemisinin–piperaquine in Africa. The latest published studies showed that PCR-corrected adequate clinical and parasitological response (APCR) at day 42 ranged between 94.6 and 100% for the treatment of uncomplicated *P. falciparum* malaria in children treated between 2011 and 2017 in Africa (Tanzania, Rwanda, Mali, Guinea, Burkina Faso, Angola, Niger) [15–20]. However, there are some rare cases of clinical failures with dihydroartemisinin–piperaquine in Africa [15]. Two cases of late treatment failure after 30 and 32 days were reported in Italian travelers returning from Ethiopia and treated with dihydroartemisinin–piperaquine [21, 22]. Additionally, some clinical failures in travelers returning from Africa, and confirmed by an expected plasmatic level of dihydroartemisinin–piperaquine, were obtained in the French national reference centre for malaria (unpublished personal data). The genes involved in resistance to artemisinin derivatives and piperaquine in Southeast Asia do not properly explain these few clinical failures observed in Africa [22–27]. It is imperative to monitor the emergence of dihydroartemisinin–piperaquine resistance in Africa. But, are *k13* and *plasmepsin II* genes reliable to survey resistance in Africa?

## Artemisinin derivative resistance

The emergence and spread of resistance to artemisinin derivatives were observed in Southeast Asia [1–6]. This resistance was associated with delayed parasite clearance half-lives (> 5 h) after artemisinin-based monotherapy treatment or ACT [1, 4, 28, 29]. Additionally, slow in vivo parasite clearance half-live was correlated with in vitro resistance manifested an increase in the ring-stage survival rate after contact with 700 nM of artemisinin for 6 h, evaluated with a new phenotypic assay, the in vitro ring-stage survival assay or RSA [30–33].

Different molecular markers, associated with in vitro resistance to artemisinin derivatives measured by standard phenotypic assays, were previously proposed. Polymorphisms in the *pfATPase6* gene, encoding the *P. falciparum* sarco-endoplasmic reticulum calcium-ATPase PfATPase 6 protein, were first associated with in vitro resistance [34], but not with in vivo delayed parasite clearance in *P. falciparum* parasites from the Thai-Cambodia border [35]. Amplification of the *P. falciparum* multidrug resistance 1 gene (*pfmdr1*) was also associated with in vitro reduced susceptibility to artemisinin derivatives [36–38], but never with delayed parasite clearance [39]. Additionally, mutations on *pfmdr1* genes were shown to be correlated with in vitro reduced susceptibility to artemisinin derivatives [40–42]. The involvement of polymorphisms in potential genes was evaluated, such *as pfubp*-*1* encoding the *P. falciparum* ubiquitin specific protease 1 [43–45], the gene encoding the RING E3 protein ubiquitin ligase [46, 47], *pfap2mu* encoding the *P. falciparum* adaptor protein complex 2 mu subunit [44, 48], *pfmdr5* encoding the *P. falciparum* multidrug resistance 5 protein [49] or *pfmdr6* encoding the *P. falciparum* multidrug resistance 6 protein [49–51]. Only mutations *pfap2mu* S160N and *pfubp1* E1525D/Q were found in cases of African imported *P. falciparum* malaria with clinical failure with ACT [25]. Whole-genome sequencing of the artemisinin-susceptible F32-Tanzania strain and the artemisinin-resistant F32-ART line, obtained after 5 years of artemisinin pressure, led to identification of several mutations (M476I, C580Y, R539T, Y493H, I543T and P574L) in the propeller domain of the *kelch 13* (*k13*) gene (PF3D7_1343700) that are associated with in vitro resistance to artemisinin [31, 52, 53]. These mutations were associated with artemisinin-resistant (high survival rate) Cambodian isolates evaluated with RSA [1, 31, 32]. Additionally, these mutations were also associated with in vivo delayed parasite clearance half-lives (> 5 h) in Southeast Asia, including Cambodia, Vietnam, Thailand, Myanmar and China [1, 31, 54] or parasitaemia still positive on day 3 after 7 days of artesunate monotherapy or 3 days of ACT [23]. Another mutation, F446I, was predominant in Myanmar and associated with high survival rate and *P. falciparum* in vivo delayed clearance [55–58].

According to the WHO, the proportion of patients still parasitaemic on day 3 (10%) or with a parasite slow clearance half-life above 5 h (10%) after artesunate monotherapy or treatment with ACT, or carrying *k13* mutations associated with artemisinin resistance in Asia are indicators to identify emergence of suspected artemisinin resistance [59]. Resistance to artemisinin is confirmed when at least 5% of the patients carry parasites with *k13* resistance-associated mutations are still parasitaemic on day 3 or show slow parasite clearance [59]. The WHO has recommended evaluate *k13* resistance-associated mutations to track emergence and spread of artemisinin resistance in Africa.

The main *k13* mutations involved in artemisinin resistance in Southeast Asia are not yet reported in Africa certainly due to an absence of artemisinin resistance in Africa [23, 60–68]. Artemisinin resistance due to *k13* mutations has not disseminated to African countries yet. However, clinical failures with ACT, although rare, were reported in Africa (Angola, Senegal, Zaire) or in imported falciparum cases from Africa (Angola, Ethiopia, Liberia, Uganda) and were not associated with *k13* resistance-associated mutations [21–25, 69–71]. In some cases, pharmacokinetic data were associated and allowed to exclude sub-therapeutic drug exposure to dihydroartemisinin [21, 22]. On Senegalese patients, parasites were still detected on day 3 after ACT treatment and were wild-type for K13 [24]. An isolate from Equatorial Guinea collected from patient with early treatment failure after artemisinin–piperaquine showed in vitro survival rate higher than the rate observed in the control strains but lower than rate in Asian artemisinin-resistant strain with a C580Y mutation [72]. However, none of the mutations described in artemisinin resistance in Asia was detected. A new mutation (M579I) was identified.

Additionally, 98.5% of Cambodian patients with isolates carrying C580Y or Y493H mutations on day 1 were negative on day 3 after dihydroartemisinin–piperaquine treatment [73]. Cambodian parasites with in vitro survival rates above the cut-off of 1% can lack the *k13* mutations involved in artemisinin resistance in Cambodia [74]. Chinese patients with R539T mutant parasites imported from Angola and P574L mutant parasites from Equatorial Guinea all recovered after treatment with dihydroartemisinin–piperaquine [75].

These data suggest that other mechanisms than *k13* mutations may explain artemisinin resistance, and more particularly in Africa. Mutations on *falcipain 2a* gene, encoding a cysteine protease and haemoglobinase and *atg18* gene, encoding the autophagy-related protein 18, might be associated with artemisinin resistance in parasites from the China Myanmar-border [76–78]. Additionally, mutations in the actin-binding protein coronin (R100K, E107V or G50E) conferred high in vitro survival rate in Senegalese *P. falciparum* strains, and this in the absence of mutation on the *k13* propeller gene [79].

## Piperaquine resistance

Emergence of *P. falciparum* resistance to dihydroartemisinin–piperaquine was observed in Cambodia, where the prevalence of recrudescent infections rapidly increased [7–9], and then in Vietnam [10, 11]. Additionally, in vitro resistance to piperaquine was detected in Cambodia and increased rapidly between 2013 and 2015 [80]. Duplication of the *Plasmodium falciparum plasmepsin II* gene (*pfpm2*) (PF3D7_1408000), encoding a protease involved in haemoglobin degradation, has been found to be associated with reduced in vitro susceptibility to piperaquine in Cambodian *P. falciparum* parasites and with dihydroartemisinin–piperaquine failures in Cambodia [81, 82]. A new in vitro test, the piperaquine survival assay (PSA), was developed to follow piperaquine resistance [83]. *Plasmodium falciparum* dihydroartemisinin–piperaquine failures in Cambodia were associated with piperaquine survival rate above 10% or high piperaquine IC_50_ above 90 nM estimated by in vitro standard assay [81–83].

However, the involvement of *pfpm2* in piperaquine resistance seems controversial in Africa. In Mali, the presence of *P. falciparum* isolates with *pfpm2* duplications was confirmed in only 7 out of 65 clinical failures with dihydroartemisinin–piperaquine [26]. Three patients harbouring parasites with two copies of *pfpm2* in Tanzania were successfully treated with dihydroartemisinin–piperaquine [84]. Additionally, only a single copy of *pfpm2* was detected in two isolates collected in imported malaria cases from Ethiopia and Cameroon after dihydroartemisinin–piperaquine failures [22, 27]. The use of dihydroartemisinin–piperaquine as intermittent preventive treatment during pregnancy did not select for *pfpm2* duplication in Uganda [85]. Ex vivo susceptibility to piperaquine in imported *P. falciparum* parasites from Africa, in Ugandan and Senegalese isolates was not associated with variation in *pfpm2* copy number ([86–88], unpublished personal data). Additionally, a recent publication showed that overexpression of *pfpm2* did not change the susceptibility of the 3D7 *P. falciparum* strain to piperaquine [89].

All these data suggest that *pfpm2* would not be the only gene that explains the resistance to piperaquine in Africa. The *P. falciparum* chloroquine resistance transporter gene (*pfcrt*) may be a causal gene because piperaquine is a dimer of chloroquine. Mutations in *pfcrt* could be involved in piperaquine resistance. However, the K76T mutation involved in chloroquine resistance was not associated with in vitro and ex vivo resistance to piperaquine [90, 91]. Novel mutations in *pfcrt*, like H97Y, F145I, M343L, C350R or G353V, seem to confer in vitro resistance to piperaquine in *P. falciparum* parasites [92–94]. However, there is no direct evidence of piperaquine inhibiting PfCRT.

## Conclusion

Mutations in K13 (C580Y, R539T, Y493H, I543T and P574L) and *pfpm2* duplications in *P. falciparum* are associated with in vitro resistance and clinical failures with dihydroartemisinin–piperaquine in Southeast Asia. Although the resistance to dihydroartemisinin–piperaquine has not yet emerged in Africa, the first data on clinical failures and in vitro reduced susceptibility suggest that *k13* and *pfpm2* would be not the only genes involved in artemisinin and piperaquine resistance. It is imperative to identify new genes to explain resistance to artemisinin and piperaquine in Africa. It is necessary to maintain tracking of the emergence and spread of *k13* and *pfpm2* mutant parasites in Africa, which could be imported from Asia. This surveillance must be associated with the tracking of dihydroartemisinin–piperaquine clinical failures in Africa due to resistant parasites. Too few studies associate drug plasmatic measures to verify good compliance and pharmacokinetic to confirm resistance. African parasites may have their own genetic background preference to select dihydroartemisinin–piperaquine resistance which surely differs from Southeast Asian parasites. These isolates should be characterized by assessing *k13* polymorphisms, *pfpm2* copy number variation, but also other potential marker of resistance. The identification of new genes involved in dihydroartemisinin–piperaquine resistance in Africa could be performed by systematic analysis of African resistant parasites by genome wide association study (GWAS).

## Data Availability

Not applicable.

## Abbreviations

ACT: artemisinin-based combination therapy
ACPR: adequate clinical and parasitological response
GWAS: genome wide association study
PCR: polymerase chain reaction
*pfap2mu*: *P. falciparum* adaptor protein complex 2 mu gene
*pfatg18*: *P. falciparum* autophagy-related gene 18
*pfATPase6*: *P. falciparum* sarco-endoplasmic reticulum calcium-ATPase 6 gene
*pfcrt*: *P. falciparum* chloroquine resistance transporter gene
*pfmdr1*: *P. falciparum* multidrug resistance 1 gene
*pfmdr5*: *P. falciparum* multidrug resistance 5 gene
*pfmdr6*: *P. falciparum* multidrug resistance 6 gene
*pfubp*-*1*: *P. falciparum* ubiquitin specific protease 1 gene
PSA: in vitro piperaquine survival assay
RSA: in vitro ring-stage survival assay
WHO: World Health Organization
